# Prognostic value of different cardiac MRI parameters for the diagnosis of myocarditis

**DOI:** 10.1186/1532-429X-13-S1-P295

**Published:** 2011-02-02

**Authors:** Waleed Ahmed, Daniel Verdini, Uthamalingam Shanmugam, Heidi S Lumish, Peerawut Deeprasertkul, Yongkasem Vorasettakarnkij, Hector M Medina, Ravi Shah, Thomas J Brady, Udo Hoffmann, Gotdfred Holmvang, Brian Ghoshhajra, David Sosnovik

**Affiliations:** 1 Massachusetts General Hospital, Boston, MA, USA

## Objective

To evaluate the prognostic utility of different cardiac MRI (CMR) parameters utilized for the diagnosis of myocarditis.

## Background

The established parameters for myocarditis by CMR include T1 global early enhancement ratio, T2 ratio and delayed enhancement (DE). Guidelines suggest that two out of three criteria should be positive for diagnosing myocarditis. The prognostic value of these criteria has not been evaluated.

## Methods

Retrospective review of all CMR cases performed for suspected myocarditis over 3 years from 7/2006 to 8/2009 was performed. MACE was defined as a composite of cardiac death, persistent NYHA class III or IV, need for ICD and cardiovascular related readmissions during the 1 year after initial presentation.

## Results

209 cases were reviewed; mean age 47 and 58% men. 74 (35%) patients were diagnosed with myocarditis based on CMR. The number of patients with 0, 1, 2 and 3 criteria positive was 70 (33%), 65 (31%), 49 (23%) and 25 (12%) respectively. Age, gender, diabetes, hypertension, smoking, CAD, prior CHF and CRI were similar among all groups. There was a trend for higher prevalence of NYHA class III or IV in myocarditis patients (17% vs 7.8 %; P=0.05). Baseline LVEF was 48.44±18 by TTE and 46±21 by CMR with no significant differences between groups. Mean increase in LVEF at 6 months by TTE for patients with 0, 1, 2 and 3 criteria positive was 5±10, 13±11, 11±10 and 6±12 (P=0.147). Mean change in LVEF for three myocarditis groups based on abnormal T1 and T2, T1 and DE or T2 and DE was 10±9, 10±7 and 7±9 (P=0.39). MACE was achieved in 20 (37%) patients with myocarditis and 21 (23%) patients without the diagnosis (P=0.078). The rate of MACE in patients with 0, 1, 2 and 3 positive criteria was 11 (22%), 10 (25%), 13 (37%) and 7 (37%) (P=0.127 and 0.21 for 0 vs 2 and 0 vs 3 criteria). The three groups based on positive T1 and T2, T1 and DE or T2 and DE had MACE rates of 3 (23%), 5 (38%) and 5 (38%) with no significant differences on pairwise comparisons.

## Conclusions

Both the number of criteria positive for myocarditis as well as any particular combination of abnormal parameters were not predictive of improvement in LVEF at 6 months or MACE upto one year. Among patients referred for CMR for suspected myocarditis the outcomes of patients with and without myocarditis by CMR were similar.

